# PReS-FINAL-2367: Academic training of pediatric rheumatologist in the Russian federation

**DOI:** 10.1186/1546-0096-11-S2-O32

**Published:** 2013-12-05

**Authors:** E Alekseeva, E Chistyakova

**Affiliations:** 1 Pediatric Faculty, I.M. Sechenov First Moscow State Medical University; 2 Rheumatology, Scientific Center of Children Health of RAMS, Moscow, Russian Federation

## Introduction

Rheumatic diseases are characterized by high prevalence and functional disability of patients. Right diagnosis in the onset of disease, early aggressive therapy with antirheumatic drugs including biologics improve the prognosis and prevent disability of patients. Only high qualified pediatric rheumatologists can do it.

## Objectives

To analyze system of pediatric rheumatologists training in Russian Federation (RF).

## Methods

Description of medical education in pediatric rheumatology in RF.

## Results

Training of pediatricians is performed at a pre-degree stage at Pediatric Faculties of State Universities. Graduates get a degree of a pediatrician.

There is no medical specialization of "Pediatric Rheumatologist" in the list of medical professions in RF. In 2012 a position of rheumatologist was included into the staff schedule of children's polyclinic. In children's hospitals Pediatric Rheumatology Departments are provided, and position of rheumatologist was included into the staff schedule of pediatric hospitals. A graduate of Pediatric Faculty or of General Medicine Faculty (with a degree of a general practitioner) serves internship in pediatrics either in the form of 1 year-internship training or in the form of 2 year-residency training. After that the graduates get a certificate of a medical specialist (pediatrician) and a right for clinical practice. Further residency training in rheumatology is required (2 years) or professional development training in rheumatology of at least 500 hour- duration. After completion the graduate receives a certificate of rheumatologist. Further training is performed in the form of advanced medical training in postgraduate stage. A rheumatologist undergoes thematic advanced training in pediatric rheumatology in the volume of 144 hours and gets a certificate. Further professional training is obtained within the system of continuous medical education.

In 2003 at the I.M. Sechenov First Moscow State Medical University a Pediatrics and Pediatric Rheumatology Chair was established, first in RF. The key area of activity is to improve qualification of pediatricians in the fields of diagnostics, treatment and rehabilitation of children with rheumatic disorders. Within 10 years 28 advanced training programs have been performed. For remote regions online programs using videoconference calls are organized. Advanced training is performed at training schools within annual Meetings of the Union of Pediatricians of RF. The School of Pediatric Rheumatologist was founded in 2006. 1,464 pediatricians took part in the School since 2006. Since 2011 precongress master classes in pediatric rheumatology have been organized within the Congress of Pediatricians of RF with participation of European and American pediatric rheumatologists. A total of 2,742 pediatricians have underwent advanced training in pediatric rheumatology in 10 years. Once in 5 years a rheumatologist should undergo training to confirm the certificate.

## Conclusion

Training of pediatricians in pediatric rheumatology is very important for suspicion of rheumatic disease at onset. Training of pediatric rheumatologists is very important for early diagnostics and adequate treatment of rheumatic diseases.

## Disclosure of interest

None declared.

